# Epidemiological Changes in Pediatric RSV Infections in Poland (2016–2024): Impact of the COVID-19 Pandemic

**DOI:** 10.3390/jcm14176284

**Published:** 2025-09-05

**Authors:** Agnieszka Blomberg, Magdalena Żak, Maria Koźba-Baranowska, Marcin Tkaczyk, Marta Depczyk-Bukała, Krzysztof Zeman

**Affiliations:** 1 Department of Pediatrics, Immunology and Nephrology, Polish Mother’s Memorial Hospital Research Institute, 93-338 Lodz, Poland; agnieszka.blomberg@iczmp.edu.pl (A.B.); krzysztof.zeman@umed.lodz.pl (K.Z.); 2 Department of Pediatrics, Immunology and Nephrology, Medical University of Lodz, 90-151 Lodz, Poland

**Keywords:** respiratory epithelial syncytial virus, RSV, COVID-19 pandemic, HFNC

## Abstract

**Objective**: The COVID-19 pandemic disrupted the seasonal pattern of RSV infections, increasing cases outside the typical epidemic season. This study aimed to assess the pandemic’s impact on the clinical characteristics of RSV infections in children hospitalized at the Polish Mother’s Memorial Health Institute in Łódź, based on a 9-year observation period from 2016 to 2024. **Methods:** A retrospective analysis was conducted on 330 children hospitalized for RSV between 2016 and 2024. Patients were divided into pre-COVID-19 (2016–2020, *n* = 160) and post-COVID-19 (2021–2024, *n* = 170) groups. The hospitalization course, the treatment duration, the use of oxygen, antibiotics, systemic steroids, the patient age structure, and risk factors were compared. **Results**: Following the pandemic, the age profile of hospitalized RSV patients shifted, with more children over 12 months (27% post-COVID-19 vs. 18% pre-COVID-19). RSV hospitalizations increased as COVID-19 cases declined (correlation coefficient: −1.0; *p* < 0.001). The average hospitalization duration decreased by 1.8 days (*p* = 0.002). Oxygen therapy was used more frequently post-pandemic (*p* < 0.001), while antibiotic use decreased (51.75% vs. 81.25%; *p* < 0.001). No significant difference was observed in ICU transfer rates. Premature infants and children with congenital defects experienced longer hospitalizations, with a stronger correlation noted post-COVID-19 (0.38; *p* < 0.001). **Conclusions**: The COVID-19 pandemic changed the profile of children hospitalized with RSV, increasing the proportion of older patients. Despite shorter hospital stays, prematurity and congenital anomalies remained key risk factors for severe disease. Study limitations include its retrospective design, selection bias, and limited socio-demographic and clinical data due to pandemic-related constraints.

## 1. Introduction

Respiratory syncytial virus is a highly contagious pathogen and the leading cause of bronchiolitis in infants and young children. Its name is derived from the virus’s ability to induce syncytia formation in the respiratory epithelium [1,2,3,4]. Usually, the course of infection is mild; however, in some cases, a severe course of the disease may develop and require hospitalization. Risk factors include prematurity, chronic respiratory disease, heart defects, neurological disease, immune disorders, an age of less than 3 months, breastfeeding for less than 2 months, nursery attendance, contact with preschool and school-age siblings, and exposure to tobacco smoke [1,2,3,5]. Most RSV infections occur in seasonal cycles. In Poland and other countries in the northern hemisphere, the RS infection season begins in October/November, reaching a peak of cases in January and ending in March/April [4,6,7,8]. However, the epidemiology and seasonality of RSV have been disrupted with the emergence of severe respiratory infections due to coronavirus 2 (SARS-CoV-2), which triggered the COVID-19 pandemic in March 2020 [9].

SARS-CoV-2 virus (severe acute respiratory syndrome coronavirus 2), causing COVID-19 acute respiratory syndrome, was isolated and began to be studied in 2019, during a global outbreak [9]. Clinical manifestations range from mild symptoms to severe pneumonia and respiratory failure. In pediatric patients, the most characteristic complication is PIMS (pediatric inflammatory multisystem syndrome temporally associated with SARS-CoV-2) [10,11,12].

The treatment of viral respiratory infections is primarily based on symptomatic therapy, and in more severe cases, oxygen therapy is required. Conventional oxygen therapy encounters certain limitations, such as the lack of precise control of the oxygen concentration in the respiratory mixture and the absence of ventilatory and gas exchange support in cases of severe respiratory failure. On the other hand, high-flow nasal cannula therapy (HFNC) is a more advanced and effective method. HFNC enables the delivery of a heated and humidified mixture of oxygen and air with a precisely determined concentration (FiO_2_) and flow rate ranging from 20 to even 60 L/min. The main advantage is the provision of minimal respiratory support by generating a small positive pressure in the airways (PEEP effect). However, despite its numerous advantages, the use of HFNC requires specialized equipment, which increases the cost of treatment significantly.

During the global COVID-19 pandemic, the implementation of non-pharmacological interventions, such as lockdowns, social isolation, mask-wearing, and the use of disinfectants in public spaces not only reduced the number of COVID-19 infections but also significantly limited the spread of other seasonal viruses, such as the influenza virus, rhinovirus, and RSV [9,13]. In Poland, these interventions were introduced in March 2020 and included nationwide school and workplace closures and limits on public gatherings. Adherence to restrictions in Poland was initially high but gradually declined as measures were eased in subsequent phases of the pandemic [12]. Once restrictions were eased, a significant increase in pathogen activity was observed, including respiratory syncytial virus. One contributing factor may have been the limited viral exposure during restrictions. Consequently, the number of children remained immunologically incompetent to RSV [1,13,14,15]. In response to the surge in RSV infections, preventive measures were introduced, including passive immunization with long-acting monoclonal antibodies and active immunization through maternal vaccination during pregnancy. These strategies open up new possibilities for protecting children from severe RSV infections and may serve as the foundation for future preventive programs [7,16,17].

It has been postulated that the COVID-19 pandemic had a profound impact on the dynamics of viral infections and the functioning of the population’s immune system. It also changed the patient profile and the clinical course of many diseases, presenting a significant challenge for modern medicine. Notably, differences in the stringency and duration of pandemic mitigation measures between countries, such as the strict, centrally enforced lockdown policies in China compared to the more moderate and regionally varied approach in Poland, may have led to distinct trajectories of viral resurgence, including the timing and intensity of RSV outbreaks.

In this study, we aimed to assess these changes and their implications for public health.

## 2. Materials and Methods

The medical records of patients with confirmed RSV infection hospitalized in the Department of Paediatrics, Immunology, and Nephrology at Mother Memorial Hospital (Instytut Centrum Zdrowia Matki Polki) between January 2016 and December 2024 were retrospectively analyzed. Patients with a diagnosis of bronchiolitis or pneumonia were included in the study. Different methods were used to confirm RSV infection: the BiofirePCR test and Fluorecare 4in1 antigen tests. The Fluorecare antigen test is a rapid immunochromatographic test designed to detect the nucleocapsid protein antigen of influenza A virus, influenza B virus, RSV virus, and SARS-CoV-2 virus. The test has 100% clinical specificity and a sensitivity of 94.35%. The BIOFIRE^®^ Respiratory 2.1 plus Panel test uses the RT-PCR method and detects 19 viruses and 4 bacteria causing upper respiratory tract infections, including RSV, with a sensitivity and specificity of 97.4% and 99.4%, respectively.

The study included pediatric patients from the neonatal period up to 5 years of age who were hospitalized with symptoms of respiratory tract infection during the study period. Only children with laboratory-confirmed RSV infection, diagnosed through antigen testing or PCR, were included in the analysis. Children older than 5 years of age and those without a confirmed RSV infection were excluded from the study. The final study population consisted of 330 children, with two distinct groups identified: 160 patients hospitalized between 2016 and 2020 (pre-COVID-19 group) and 170 patients hospitalized between 2021 and 2024 (post-COVID-19 group).

Cases from both the pandemic period and the post-epidemic phase were classified as part of the post-COVID-19 group for statistical analysis. Given the seasonal occurrence of RSV cases between December and March, and the official onset of the COVID-19 pandemic on 20 March 2020, cases identified in 2020 were classified as pre-COVID-19 for the purposes of this study.

The participation rate was 100%, as all patients hospitalized with RSV infection during the study period were included in the analysis.

The study analyzed the course of hospitalization in terms of variables such as the duration of a hospital stay, the need for oxygen therapy, and antibiotic therapy. In addition, the age structure of the study populations and selected risk factors for more severe RSV, including prematurity and coexisting congenital malformations, were assessed.

The Shapiro–Wilk test was used to assess the normality of the data distribution, which showed that the data were not normally distributed. Therefore, non-parametric tests were applied for statistical analyses. Due to the lack of normality, median values and interquartile ranges (IQRs) were used instead of means. Spearman’s method was used to calculate correlations, and the Chi-square test was employed to compare data distributions. A *p*-value of 0.05 was considered the threshold for statistical significance.

Calculations were performed using scripts written in Python (v.3.10) in Python (v.6.5.4, Project Jupyter, Berkeley, CA, USA) with the following libraries: Pandas (v.2.0.3), Numpy (v.1.25.2), Math, Matplotlib (v.3.8.4), Scipy (v.1.12.0), Seaborn (v.0.13.2), and Pylab (v.3.8.4).

## 3. Results

**Figure 1:**
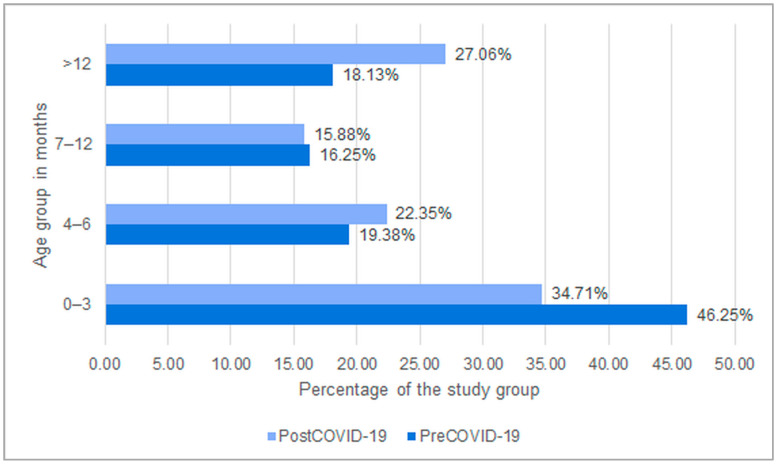
Age distribution of children hospitalized due to RSV infection in the pre-COVID-19 and post-COVID-19 groups.

A retrospective analysis of medical records was conducted for 330 patients. The pre-COVID-19 group consisted of 160 patients, including 73 girls (45.6%) and 87 boys (54.4%). The median age of this population was 4 months (IQR 7 months). Following the outbreak of the SARS-CoV-2 pandemic, 170 children were hospitalized in the post-COVID-19 group, including 60 girls (35.3%) and 110 boys (64.7%), with a median age of 6 months (IQR 11.75 months). No statistically significant differences in gender distribution were observed between the groups (*p* = 0.072). In both groups, the majority of patients were under 12 months of age, accounting for around 82% in the pre-COVID-19 group and 73% in the post-COVID-19 group. The largest proportion of children were aged up to 3 months, with 46.3% before the pandemic and 34.7% after it. A statistically significant difference in age distribution was observed between the two groups, with an increase in the number of children over 12 months of age in the post-COVID-19 group (Figure 1).

A summary of patient characteristics and the statistical significance of between-group differences is presented in Table 1.

**Figure 2:**
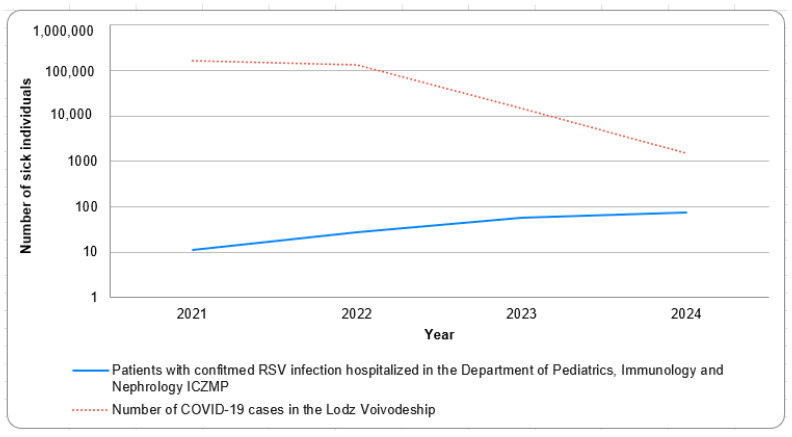
The relationship between the number of children hospitalized due to RSV infection and the number of COVID-19 cases in the Łódź Voivodeship.

The relationship between the number of children hospitalized for RSV and the number of new COVID-19 cases in the Łódź region was analyzed, revealing that the number of RSV-related hospitalizations was lowest during the first infection season immediately following the COVID-19 pandemic outbreak, gradually increasing in the subsequent years (Figure 2). It was observed that the decrease in COVID-19 cases was correlated with an increase in RSV infections (correlation coefficient −1.0; *p*-value < 0.001).

**Figure 3:**
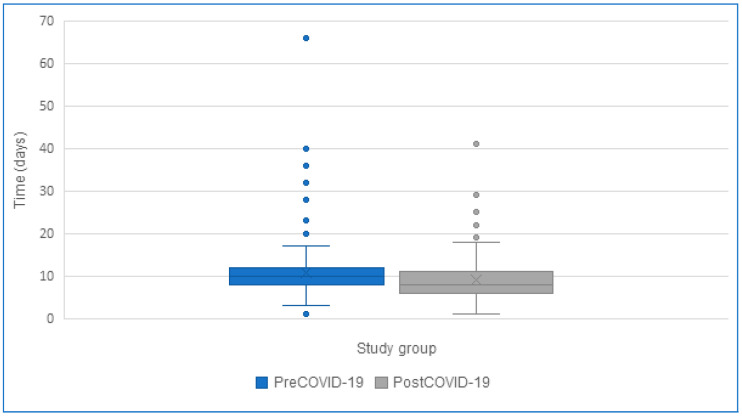
Length of hospitalization of patients hospitalized due to RSV infection.

Regarding the length of hospitalization, the average duration in the pre-COVID-19 group was 10.9 days (SD 6.8), which was longer compared to the post-COVID-19 group, where the average was 9.1 days (SD 5.4). The median length of hospitalization was 10 days (IQR 4) in the pre-COVID-19 group and 8 days (IQR 4.75) in the post-COVID-19 group (*p* = 0.002) (Figure 3).

No statistically significant relationship between the length of hospitalization and age was found in the post versus pre-COVID-19 period (Table 1). The average length of hospitalization was similar across all age groups. However, the analysis revealed a statistically significant difference in the length of hospitalization for the groups aged >12 months and 7–12 months (Table 2).

A trend towards shorter hospitalization times was also observed in other age groups, although it did not reach statistical significance. The use of oxygen therapy in the post-COVID-19 group significantly contributed to a longer hospital stay (*p* < 0.001), a finding not observed in the pre-COVID-19 group.

**Figure 4:**
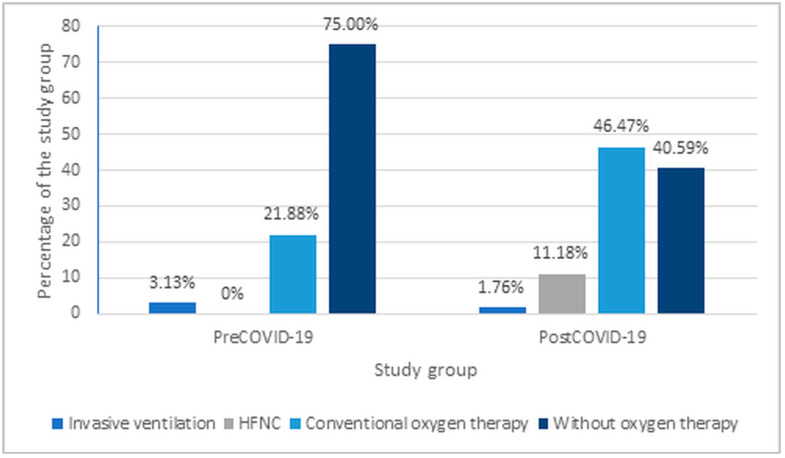
Oxygen therapy methods used in patients hospitalized due to RSV infection.

An analysis of treatment methods for RSV infections was performed, including oxygen therapy. It was observed that oxygen therapy was significantly less frequent in the pre-COVID-19 group compared to the post-COVID-19 group (*p* < 0.001) (Figure 4).

In the post-COVID-19 group, oxygen therapy was significantly associated with longer hospital stays (*p* < 0.001), a relationship that was not demonstrated in the pre-COVID-19 group.

**Figure 5:**
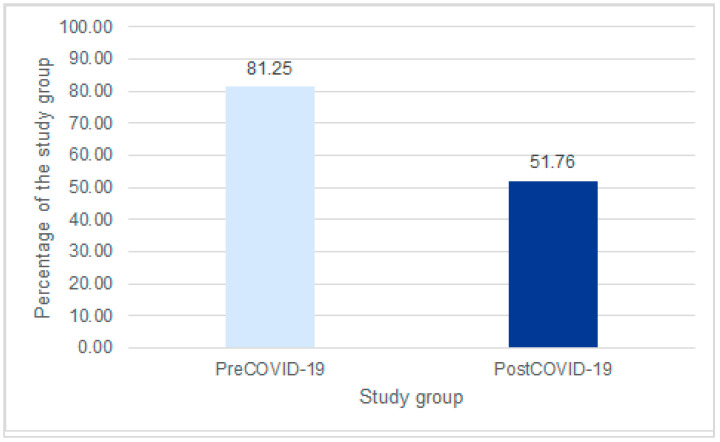
Use of antibiotics in patients hospitalized due to RSV infection.

In cases where RSV complications were identified or suspected, such as pneumonia, otitis media, or co-infection with Mycoplasma pneumoniae, antibiotic therapy was administered. A significantly higher proportion of patients in the pre-COVID-19 group received antibiotics (81.25%) compared to the post-COVID-19 group (51.75%) (*p* < 0.001) (Figure 5).

Treatment failure in the pediatric ward, requiring patient transfer to the intensive care unit, occurred for 3.1% of patients in the pre-COVID-19 group and 1.8% in the post-COVID-19 group. No statistically significant difference was found between these data.

The analysis also included specific patient groups at higher risk for severe RSV infection. The first group consisted of premature infants, which made up 10% of the pre-COVID-19 group. In this group, the median length of hospitalization was the same for both premature infants and full-term infants—10 days (IQR 4). In the post-COVID-19 group, 4.7% of the patients were born prematurely. The median length of hospitalization for these children was 12.5 days (IQR 4.5), whereas for full-term infants, the median was 8 days (IQR 4). All of the premature infants in both the pre-COVID-19 and post-COVID-19 groups required antibiotic therapy. Oxygen therapy was necessary for 35% of premature infants in the pre-COVID-19 group, while it was required for 100% of premature infants in the post-COVID-19 group. RSV prophylaxis with palivizumab was administered to two patients in the pre-COVID-19 group and four patients in the post-COVID-19 group, which represented 12% and 50% of premature infants in each respective group.

Another group of children analyzed were those with congenital defects that significantly affect respiratory and immune system function (in our group, these included heart defects, respiratory system defects, and congenital immunodeficiencies). In the pre-COVID-19 group, 14 children were diagnosed with congenital defects, representing 8.75% of the study population. The median hospitalization duration for this group was 10.5 days (IQR 4.75), with an average of 18.21 days (SD 16.7). Children hospitalized during the same period without a history of congenital defects had an average hospital stay of 10.21 days (SD 4.56), with a median of 10 days (IQR 4). In the post-COVID-19 group, 15 children had a positive medical history for congenital defects, representing 8.82% of the population. The average hospitalization duration for this group was 17 days (SD 10.6), with a median of 13 days (IQR 14). In contrast, children without congenital defects had an average hospital stay of 8.38 days (SD 3.8), with a median of 8 days (IQR 4).

Considering variables such as concomitant prematurity, congenital defects, and the absence of additional medical burdens, a Spearman correlation test was performed. For the pre-COVID-19 group, a positive correlation was found between the length of hospitalization and the presence of congenital defects in children hospitalized due to RSV infection, at a level of 0.18 (*p* = 0.021). For the post-COVID-19 group, a positive correlation was observed between the length of hospitalization and the presence of congenital defects, at a level of 0.38 (*p* < 0.001), while a negative correlation was found for the absence of additional burdens (such as prematurity or congenital defects), at a level of −0.28 (*p* < 0.001).

## 4. Discussion

Epidemiological changes caused by the emergence of a new variant of the SARS virus in 2019 have prompted research into the transmission dynamics and severity of multiple viral diseases [14,18,19,20]. We analyzed infections with RSV etiology in pediatric patients. Our results correlate with observed epidemiological changes in the literature, showing a deceleration in the number of RSV infections during the most severe stage of the COVID-19 epidemic and a change in the profile of patients hospitalized for RSV infections [6,21,22].

The analysis conducted, similar to the studies by S. Maslowski, Fushli, and Hai-feng [6,21,22], showed a change in the age distribution of children hospitalized for RSV infections since the start of the COVID-19 pandemic. A greater age dispersion was found, and infections occurred more frequently in the group of children older than 12 months. It can be suspected that the reason for the observed correlations was the use of isolation and personal protective measures, which led to a limitation of RS virus exposure in children born during the pandemic [13]. It also cannot be excluded that, due to the popularization of antigen tests used to confirm viral infections (RSV, influenza, COVID-19), RSV infections were more frequently diagnosed in children >12 months of age [7,14,20]. In the pre-pandemic period, access to PCR testing for respiratory pathogens, including RSV, was limited. The COVID-19 pandemic led to a broader implementation of molecular and antigen-based diagnostics, increasing detection rates of RSV in the post-COVID-19 group.

The study proved that the lowest number of hospitalizations and, therefore, RSV activity was recorded in the first years of the pandemic. In the following years, a gradual increase in the number of hospitalizations due to RSV infection was observed. Similar observations were made by Lastrucci and Hai-Feng [22,23]. The reasons for this are mainly considered to be the widespread use of personal protective equipment and social isolation [19,22].

A further analysis showed that the decrease in COVID-19 cases was accompanied by an increase in the incidence of RSV infection. These results are consistent with the observations of researchers from the Military Medical Institute in Warsaw in 2022 [1], who described the phenomenon of a so-called compensatory epidemic. It manifested in a change in the epidemic season and a significant increase in the number of RSV infections in 2021, exceeding previous forecasts. The most commonly cited reason for this trend is a weakening of collective immunity and an increase in the proportion of susceptible individuals. Similar observations over the same period have also been reported in Western Australia, the United States, Japan, and Switzerland [21,24,25,26].

An important observation from the data analysis is that the average length of hospitalization in the post-COVID-19 group was reduced by more than 1.5 days compared to the pre-pandemic period. Corresponding results were obtained in the German population in a study conducted by S. Maslowski [6]. Improvements in the quality of care for patients with viral infections, which were developed during the COVID-19 pandemic and implemented in the treatment of children with RSV infections, were suggested as a likely reason for this change. It is significant to note that the average hospitalization time for RSV-infected children in Germany was more than twice as short as in the present study, both in the pre-COVID-19 and post-COVID-19 groups [6]. This finding points to a need for a further improvement of care for patients hospitalized for RSV infections in Poland and an increased role of primary care physicians in the management of these cases. It should also be mentioned that the impact of the pandemic on the length of hospitalization was not confirmed by Dr. Lastrucci in a study conducted in Italy, where the average length of hospital stay was 5 days [22]. This period was significantly shorter than the one observed in the present study (10 days vs. 5 days).

According to the American Academy of Pediatrics, the standard treatment for viral bronchiolitis is oxygen therapy (in case of hypoxaemia) and hydration. Bronchodilators and glucocorticosteroids should not be used routinely. Respiratory physiotherapy is also not routinely recommended [27]. Depending on the patient’s clinical condition, both conventional oxygen therapy and high-flow ventilation therapy (HFNC) are used. This comparative analysis showed a significant increase in the use of oxygen therapy in the post-pandemic period (post-COVID-19) compared with the pre-pandemic period (22.5% vs 57.6%).

The obtained results are consistent with the observations of S. Maslowski et al., which demonstrated an increased demand for mechanical ventilation in the post-COVID-19 period, despite no changes in the number of admissions to intensive care units. The authors suggest that this may have been a consequence of the more frequent implementation of high-flow oxygen therapy outside intensive care units, which had not previously been a standard [6]. Similar trends were also noted in a Korean multicenter study involving children aged 3 to 24 months. During the 2021/2022 epidemic season, a significant increase in the percentage of patients requiring respiratory support (19.2%) was observed compared to previous RSV infection seasons (1.8% in the 2017/2018 season and 6.0% in 2019/2020; *p* = 0.012) [28]. On the other hand, the analysis conducted by V. Lastrucci did not confirm differences in the frequency of oxygen therapy use between groups of patients hospitalized before and after the pandemic [22].

In the Pediatric Clinic conducting this analysis, the availability of HFNC therapy significantly increased during the pandemic due to enhanced clinical awareness and the acquisition of appropriate equipment. As a result, most children requiring advanced respiratory support could be effectively managed within the general pediatric ward, reducing the need for transfer to the ICU.

Considering the demand for intensive care unit (ICU) stays among children, the findings of S. Masłowski (2024) and Lastrucci (2024), in line with our observations, did not show statistically significant differences in the number of pediatric patients requiring intensive medical care due to RSV infection [6,22]. The mentioned authors emphasize the importance of high-flow nasal cannula therapy. In contrast, the results of a retrospective, multicenter study conducted by Movvy and colleagues indicate a higher percentage of admissions to neonatal and infant (<1 year old) intensive care units compared to the pre-pandemic period (69% vs. 60%) [29].

According to the guidelines of the National Antibiotic Protection Program (NPOA), symptomatic treatment is recommended for RSV infections [30]. Despite the lack of indications for routine antibiotic use, literature data indicate that antibiotics are administered in 34–99% of RSV infections in children, most commonly for the prevention or treatment of bacterial superinfection [17,19,31,32].

In a Swiss study by K. Fischli and colleagues, a stable proportion of approximately 25% of RSV patients received antibiotic therapy in the years 2023–2024. The most common indications for its use were confirmed bacterial superinfections and complications such as acute otitis media (9.9%) and pneumonia (7.9%) [17,21,31]. On the other hand, in the study analyzed by M. Pogonowska et al. from the Warsaw Medical Institute in Warsaw, all hospitalized children (100%) in the 2020–2021 period received antibiotic therapy after confirmation of inflammatory changes in the lungs or in cases of deterioration of general condition with no improvement after symptomatic treatment. In our study group, the percentage of children receiving antibiotic therapy was 81.25% in the pre-COVID-19 pandemic period and 51.76% in the post-COVID-19 group. The most common reason for antibiotic administration was confirmed or suspected bacterial superinfection accompanying RSV infection. The observed reduction in the frequency of antibiotic use in the post-pandemic period can be related to intensified educational efforts about antibiotic resistance and reducing the overuse of antibacterial drugs. A significant factor was also the change in therapeutic approaches after the COVID-19 pandemic and the increased effectiveness of symptomatic treatment, which helped minimize the need for antibiotics in children with RSV infections.

Congenital heart defects and neuromuscular diseases were significant independent risk factors for ICU hospitalization and antibiotic therapy during RSV infections [5,7,17,21]. Similar observations were made by Hai-Feng Liu’s team from China, who identified an early age, a low birth weight, prematurity, an atopic history, general condition, and elevated IL-6 levels as common independent risk factors for severe lower respiratory tract infections both before and after the COVID-19 pandemic. However, seizures and coexisting chronic diseases were significant risk factors exclusively in the post-COVID-19 group [23]. Meanwhile, S. Masłowski’s study demonstrated a comparable percentage of hospitalized preterm infants in the pre-pandemic and post-pandemic periods [6].

Recent advances in RSV prevention have introduced promising strategies with significant public health implications. In 2023, Catalonia (Spain) launched a regional immunization program using nirsevimab, a long-acting monoclonal antibody, demonstrating a marked reduction in RSV-related hospitalizations, ICU admissions, and primary care visits [33,34,35]. The same year, the EMA approved two RSV vaccines, Arexvy (for patients aged ≥60 years) and Abrysvo (for individuals aged ≥60 years and pregnant women), which enable the passive protection of newborns through transplacental antibody transfer, providing protection against severe respiratory infections up to six months of age [27,36,37]. In Poland, palivizumab has been used since 2008 for high-risk infants, and eligibility was expanded in 2018 [38,39,40].

Epidemiological data from Northern Italy [41] highlight the significant health and economic burden that RSV-associated lower respiratory tract infections impose on healthcare systems, particularly in infants under six months of age. Universal immunization with nirsevimab in that context was projected to significantly reduce healthcare costs. However, due to the current lack of widespread access to nirsevimab in Poland, a comparable analysis of its impact cannot yet be conducted locally [42].

Due to the growing importance of RSV prevention, the World Health Organization has recognized it as one of the global health priorities. This is particularly crucial not only for the pediatric population but also for adults, especially in the context of an aging society and the burden of multiple comorbidities. In Poland, the Polish Pediatric Society, in collaboration with national experts in neonatology, vaccinology, and family medicine, has developed the health prevention program for 2025–2030. This effort aims to provide universal access to nirsevimab for all infants, regardless of the timing of their birth [36].

This study involved several limitations that should be acknowledged. First, the analysis included only hospitalized children aged 0–5 years with laboratory-confirmed RSV infection, which may introduce selection bias and limit the generalizability of the findings to milder, community-managed cases. Second, changes in diagnostic practices over time—particularly the increased availability and use of antigen and PCR testing in the post-pandemic period—may have influenced case detection rates and contributed to observed differences between study periods. Finally, due to the retrospective design and constraints related to data collection during the COVID-19 pandemic, it was not possible to fully adjust for all potential confounding factors, such as detailed socio-demographic variables (e.g., maternal age, presence of siblings, daycare attendance), birth history (e.g., birth weight), and comorbidities. These limitations may have impacted the interpretation of differences observed between the pre- and post-COVID-19 groups.

## 5. Conclusions

The analysis indicates that the COVID-19 pandemic has changed the profile of children hospitalized for RSV infection. Currently, older children, over 7 months of age, are more frequently affected by this disease. Additionally, the length of hospitalization has decreased, which is a result of advancements in oxygen therapy, symptomatic treatment, and the increasing awareness of medical personnel regarding current standards for treating viral infections, stemming from educational initiatives. Notably, no impact of the pandemic on the severity of RSV infections, particularly regarding the need for intensive care treatment, was observed. However, prematurity and congenital defects remain significant risk factors for a more severe course of the disease.

Policy-makers should enhance funding for RSV prevention and treatment programs, promote standardized clinical guidelines, and focus on protecting vulnerable populations, including preterm and medically complex children.

Given the limitations of our study—such as its retrospective design and incomplete availability of certain clinical and socio-demographic data—this issue warrants further investigation. To address these gaps, we plan to continue the prospective observation of this patient group to better understand the evolving epidemiology and risk factors of RSV infection.

## Figures and Tables

**Table 1:** Pre- and post-COVID-19 groups—patient characteristics.

Characteristic	Pre-COVID-19	Post-COVID-19	*p*-Value
Total number of patients	160	170	-
Age (Median age)	4 months	6 months	<0.001
Age Stratification	0–3 months: 46%,	0–3 months: 35%,	0.947
	4–6 months: 19%,	4–6 months: 22%,	0.971
	7–12 months: 16%.	7–12 months: 16%.	1.0
	>12 months: 18%	>12 months: 18%	1.0
Gender (Male/Female)	87 (54.4%)/73 (45.6%)	110 (64.7%)/60 (35.3%)	0.072
Length of Hospital Stay (Median)	10 days	8 days	<0.001

**Table 2:** Length of hospitalization by age in pre- and post-COVID-19 groups.

Age (Months)	Average Length of Hospitalization (Days) in the Pre-COVID-19 Group	Average Length of Hospitalization (Days) in the Post-COVID-19 Group	Statistical Significance
0–3	10.314815	8.932203	*p* = 0.052
4–6	11.837838	9.868421	*p* = 0.086
7–12	11.884615	9.370370	*p* = 0.012
>12	10.279070	8.695652	*p* = 0.007

## Data Availability

Data are available from the corresponding author upon reasonable request. Due to legal and ethical constraints under Polish law and GDPR, the dataset, which includes anonymized data from minors, cannot be made publicly available. Access may be granted under data use agreements ensuring compliance with applicable regulations.

## Abbreviations

The following abbreviations are used in this manuscript:

HFNC	High-flow nasal cannula therapy
ICU	Intensive care unit
PIMS	Pediatric inflammatory multisystem syndrome temporally associated with SARS-CoV-2
EMA	The European Medicines Agency
